# Emerging applications of Zintl clusters in small molecule activation and catalysis

**DOI:** 10.1039/d6sc05790j

**Published:** 2026-10-21

**Authors:** Bono van IJzendoorn, John S. Wenger, Meera Mehta

**Affiliations:** a Department of Chemistry, University of Oxford 12 Mansfield Road Oxford OX1 3QR UK bono.vanijzendoorn@chem.ox.ac.uk meera.mehta@chem.ox.ac.uk

## Abstract

Zintl clusters and phases have long served as model systems for exploring unusual bonding, electronic structures, and solution-state reactivity at the interface of molecular and solid-state chemistry. More recently, advances in cluster synthesis and functionalisation have enabled the emergence of Zintl-based systems capable of mediating a diverse range of catalytic transformations and small molecule activation processes. These developments have established Zintl species as versatile platforms that can function as ligands and supports for transition metal catalysts, non-innocent frameworks that actively participate in substrate activation, and heterogeneous catalytic materials. In this Perspective, we summarise recent progress in both transition metal and transition metal-free Zintl catalysis, highlighting the mechanistic diversity and design principles that underpin their reactivity. We discuss how features such as structural flexibility, cooperative reactivity, redox activity, and precise synthetic tunability have allowed these Zintl materials to be developed as catalyst platforms, and consider future opportunities for development in the field.

## Abstract 2

Emerging field of Zintl cluster catalysis; from curiosities to impactful transformations.

## Graphical abstract

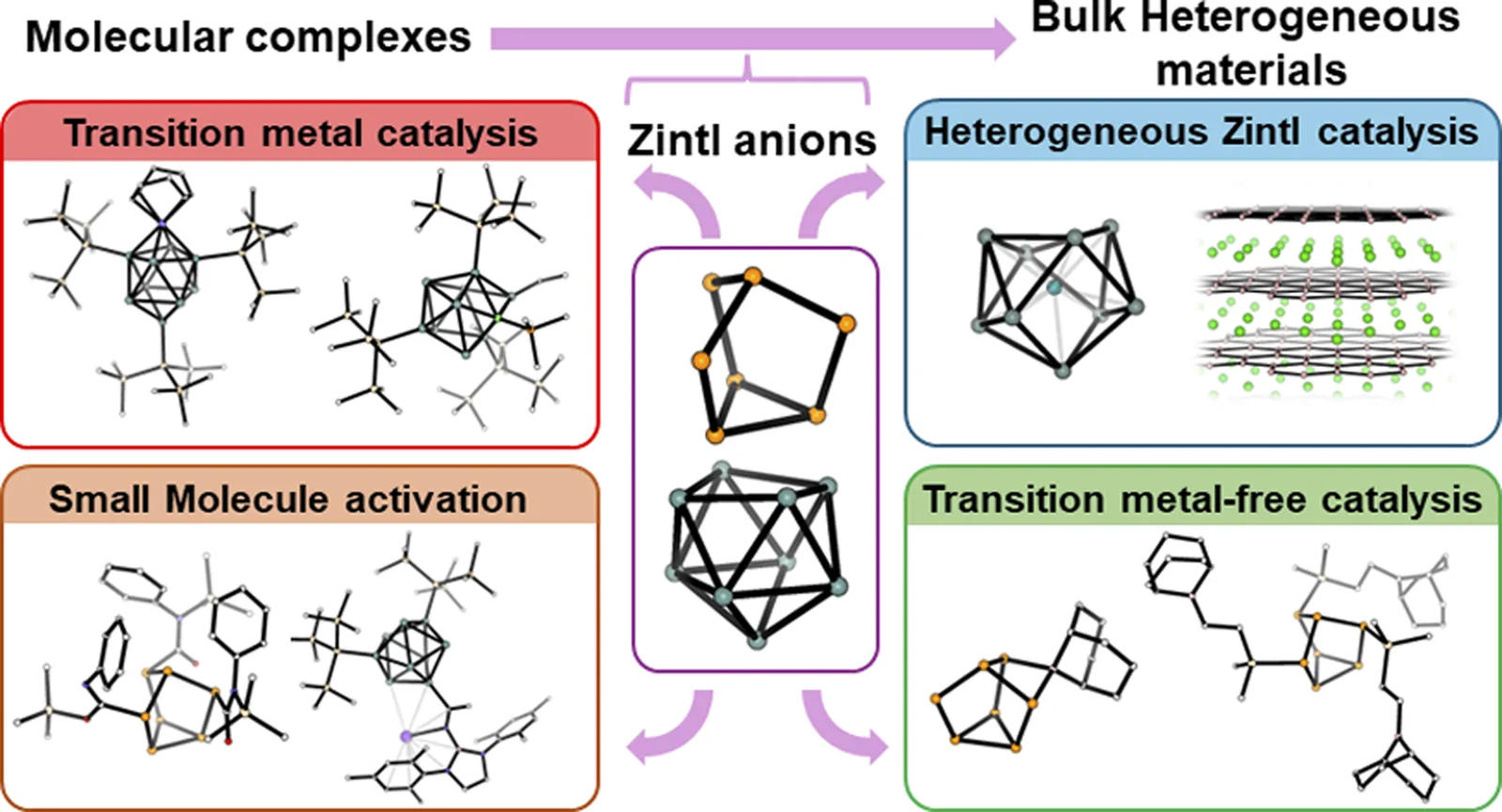

## Introduction

Zintl phases are intermetallic solid-state materials comprising electropositive metals and more electronegative p-block elements, in which formal electron transfer gives rise to p-block anions.^1^ Zintl phases are a large class of compounds and are often completely insoluble materials. Some of these Zintl phases however can be dissolved in highly polar solvents, and this is often facilitated by cation-sequestering agents such as crown ethers or cryptands. Dissolution of these soluble phases can yield discrete molecular anions known as Zintl ions or Zintl clusters.^2,3^ Although Zintl clusters are often accessed *via* their respective Zintl phases, certain Zintl clusters can also be prepared from molecular precursors; for example, the heptaphosphide anion [P_7_]^3−^ is also accessible from P_4_ and LiPH_2_.^4^ These Zintl clusters exhibit diverse geometries, unusual electronic structures, and rich solution-state reactivity. Owing to their unique position at the interface of molecular and solid-state chemistry, Zintl clusters can be regarded as soluble, atomically precise models of extended materials that are otherwise difficult to study at the molecular level.^5^ For example, the heptaphosphide cluster [P_7_]^3−^ may be viewed as a molecular fragment of red or violet phosphorus, while transition metal-functionalised [Ge_9_] clusters have been proposed as molecular models for transition metal-main group alloys.^6–12^ Such features have made Zintl clusters attractive platforms for fundamental studies of bonding, structure, and reactivity, while also providing opportunities for the development of new applications, including within catalysis.

Since their discovery, Zintl clusters have attracted considerable attention owing to their unusual structures and electronic properties. Early studies were primarily concerned with their synthesis, extraction and characterisation, understanding their bonding and electron delocalisation, and their relationship to extended solid-state materials.^1^ Subsequent advances in synthesis and derivatisation enabled increasingly sophisticated investigations of their solution-state chemistry, including redox processes, Brønsted acid-base chemistry, fluxional behaviour in solution, cluster fragmentation, reactivity towards organic substrates, and coordination to transition metals.^1,3,13–18^ These studies demonstrated that Zintl clusters can support chemical phenomena that are often difficult to access in conventional molecular systems, including three-dimensional aromaticity and antiaromaticity, and unusual magnetic properties.^19–24^ In parallel, their well-defined compositions and controlled reactivity have enabled their use as molecular precursors to functional materials, including semiconductor nanostructures and light-emitting devices.^25,26^ Despite these advances, the exploitation of Zintl clusters in catalytic transformations remained comparatively rare.

Heterogeneous catalysts and nanomaterials dominate many industrially important transformations owing to their robustness, scalability, and ease of separation; however, identifying the nature of active sites and reaction pathways in these systems often remains challenging.^27–30^ Conversely, molecular catalysts benefit from precise structural definition and mechanistic accessibility but frequently lack direct relevance to extended materials.^7^ Zintl clusters offer an attractive opportunity to bridge this divide by providing structurally well-defined and readily characterisable platforms while maintaining a closer relationship to solid-state materials. In addition, their electron-rich frameworks often exhibit accessible redox chemistry, while the presence of multiple proximal reactive sites and structural flexibility can facilitate small molecule activation and cooperative bond activation processes. Combined with the ability to selectively functionalise cluster surfaces with transition metals and main group substituents, these characteristics provide opportunities for the development of well-defined catalytic platforms relevant to both homogeneous and heterogeneous catalysis.

Over the past decade, advances in cluster synthesis, functionalisation, and mechanistic characterisation have enabled the emergence of Zintl-based systems capable of mediating a growing range of catalytic and small-molecule activation processes. These studies have revealed that Zintl clusters can participate in catalysis through several fundamentally different modes of action, including as ligands and supports for transition metal and main group catalysts, as non-innocent components for substrate activation, and as redox-active frameworks. In this perspective, we highlight recent developments in the use of Zintl clusters and phases for small molecule activation and catalysis, identify emerging design principles that underpin their reactivity, and discuss opportunities for future development.

### Transition metal-functionalised Zintl clusters in homogeneous catalysis

As Zintl clusters are electron-rich by nature and often possess multiple charged vertices, they exhibit a range of different coordination modes to transition metals, and consequently a large family of transition metal Zintl cluster coordination complexes have been reported.^1,3,15,16,18,31,32^ Incorporation of transition metals onto Zintl clusters for catalysis has been described previously as molecular analogues for single-site heterogeneous catalysts (SSHCs), which are widely used in various chemical industries.^29,33–35^ In SSHCs the metal is usually supported by heterogeneous carriers, which comes with several challenges in studying SSHCs as a consequence. Substituting the heterogeneous carrier for a soluble homogeneous Zintl cluster has certain benefits, including: (1) enabling *in situ* monitoring using solution-state methods, like nuclear magnetic resonance (NMR), Ultraviolet-visible (UV-Vis), Electron Paramagnetic Resonance (EPR) spectroscopy; (2) well defined and identical metal reactive sites throughout the material; and (3) reduced aggregation of metals due to strong interaction with the Zintl cluster.

Within homogeneous transition metal Zintl catalysis, the tetrel [E_9_]^4−^ clusters (E = Si, Ge, Sn, Pb) have been the most extensively studied. This family of clusters can be accessed *via* high temperature reactions of the respective Group 14 elements with alkali metals, usually potassium, producing a binary phase.^36–38^ From this phase, [E_9_]^4−^ anions can be extracted with the s-block cation sequestered or the [E_9_]^4−^ cluster can be functionalised with either a main group moiety or transition metal starting directly from the phase or extracted polyanionic salt.^36,39^ Most relevant to the design of these cluster platforms in catalysis, a range of silylated [Ge_9_] clusters are known with the general molecular formula [(R_3_Si)_3_Ge_9_]^−^. Of these silylated clusters, the [Hyp_3_Ge_9_]^−^ (Hyp = tris(trimethylsilyl)silyl) cluster often serves as a starting point to transition metal Zintl catalyst design due to its high solubility in common laboratory solvents.^40–44^ A range of different coordination modes are reported for transition metal-[(R_3_Si)_3_Ge_9_] complexes (Fig. 1). For example, the η^1^-coordinated complexes [(Hyp_3_Ge_9_)Cr(CO)_5_]^−^, [((^*t*^Bu_2_HSi)_3_Ge_9_)RuCp(PPh_3_)_2_], and [(Hyp_3_Ge_9_)TiCp_2_(MeCN)] (Cp = cyclopentadienyl; MeCN = acetonitrile) have been reported.^45–48^ η^3^-Coordinated complexes are also known: monomeric complexes include [(Hyp_3_Ge_9_)M(NHC^Dipp^)] (M = Cu, Ag, Au) (NHC^Dipp^ = 1,3-bis(2,6-diisopropylphenyl)imidazole-2-ylidene) and [(Hyp_3_Ge_9_)M(L)] (M = Ni, L = 1,2-bis(diphenylphosphino)ethane (dppe); M = Zn, L = pentamethylcyclopentadienyl (Cp*); M = Cu, L = P^*i*^Pr_3_; M = Rh, L = PMe_3_, PPh_3_, 1,3,4,5-tetramethylimidazol-2-ylidene (IMe_4_)); dimeric complexes include [(Hyp_3_Ge_9_)M(Hyp_3_Ge_9_)]^*x*−^ (M/*x* = Mn/0, Pd/2, Cu/1, Au/1, Ag/1, Zn/0, Cd/0, Hg/0) and [HypZn(Hyp_3_Ge_9_)Pt(Hyp_3_Ge_9_)ZnHyp]; cationic complexes include [(Hyp_3_Ge_9_){Ni(dppe)_2_}_2_]^+^.^40,49–55^ The η^4^-coordinated [(Hyp_3_Ge_9_)Cu(NHC^*i*Pr^)] (NHC^*i*Pr^ = 1,3-diisopropylimidazolylidene) and [(Hyp_2_Ge_9_)M(NHC^R^)]^−^ (M = Cu, NHC^R^ = NHC^Dipp^, NHC^*i*Pr^, 1,3-dimesitylimidazolylidene (NHC^Mes^)); (M = Ag, NHC^R^ = NHC^Dipp^; M = Au, NHC^R^ = NHC^Dipp^) have been reported. Further, the η^5^-coordinated complexes [(Hyp_3_Ge_9_)M(CO)_3_]^−^ (M = Cr, Mo, W); [(Hyp_3_Ge_9_)Rh(L)] (L = dppe, pentamethylcyclopentadienyl (Cp*)); [(Hyp_3_Ge_9_)M(PR_3_)] (M = Pd or Pt, R = Ph; M = Ni, R = Ph, 4-tolyl, ^*i*^Pr, Me) are known.^46,56–60^ Although all the complexes listed above have not been studied in their catalytic applications, and although not a comprehensive list of all the coordination complexes reported with the [Ge_9_] framework, these examples (and the ones listed below) highlight the diverse binding modes Zintl clusters have to offer.

**Fig. 1:**
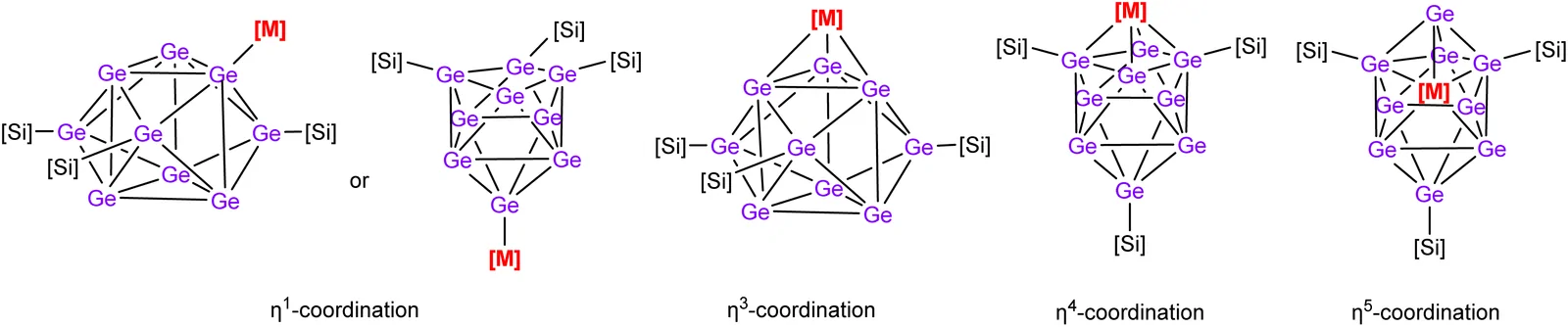
Common transition metal coordination modes to silylated [Ge_9_] clusters.

One of the first η^4^-coordinated transition metal [Hyp_3_Ge_9_] complexes reported was [(Hyp_3_Ge_9_)Rh(COD)] (COD = 1,5-cyclooctadiene) (1), which was also the first of these complexes probed for its catalytic activity (Fig. 2a).^12^ The synthesis of [(Hyp_3_Ge_9_)Rh(COD)] was reported by Goicoechea, Weller and co-workers in 2020 and was further employed in the catalytic hydrogenation of COD and *cis*-cyclooctene (COE), Fig. 2b. As mentioned before, the [Hyp_3_Ge_9_]^−^ is coordinated to the Rh-centre *via* an η^4^-coordination mode, while the COD ligand is also η^4^-coordinated, Fig. 2a. The proposed hydrogenation mechanism starts from [(Hyp_3_Ge_9_)Rh(COD)] with a η^4^–η^2^ dissociation-step of the COD ligand. The resulting 16-electron Rh(i) reversibly binds H_2_ forming a Rh(iii) dihydride species, Fig. 2c. Reductive coupling *via* an alkyl hydride species allows for the formation of the Rh(COE) complex and the COE ligand is further hydrogenated in a similar fashion. Experimental kinetic and computational studies indicate significantly faster hydrogenation of COE over COD. Heterogeneous catalysis was discounted as the reaction rate was unaffected by the addition of Hg.

**Fig. 2:**
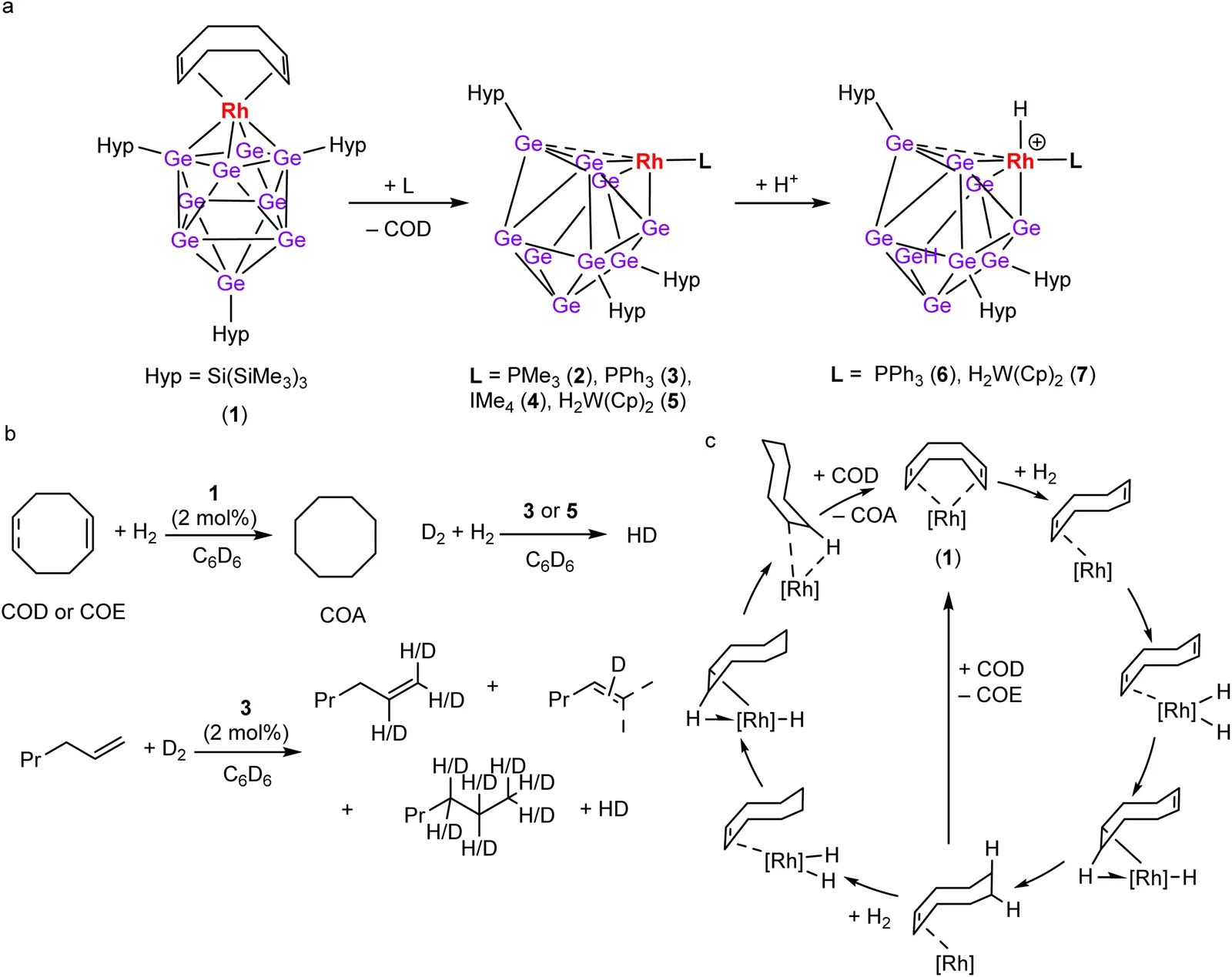
(a) Catalysts 1–7. (b) Catalytic hydrogenation and hydrogen isotope exchange of alkenes. (c) Mechanism of COD hydrogenation.

Following on from this work in 2022, the same authors reported ligand exchange reactions of [(Hyp_3_Ge_9_)Rh(COD)] (1) with the L-type ligands PMe_3_, PPh_3_, IMe_4_ and [W(Cp)_2_H_2_], which gave coordinatively unsaturated trigonal pyramidal Rh(i) centres [(Hyp_3_Ge_9_)Rh(L)] (2–5) (Fig. 2a).^50^ Unlike compound 1, clusters 2–5 were found to be unreactive toward alkenes and computed molecular orbitals revealed the highest occupied molecular orbital (HOMO) was largely located on the Rh centre. To probe this HOMO character, compounds 3 and 5 were reacted with Brookhart's acid, [H(OEt_2_)_2_][BAr^F^_4_] (Ar^F^ = 3,5-(CF_3_)_2_C_6_H_3_), and afforded the respective protonated clusters 6 and 7 (Fig. 2a). Further, clusters 3 and 5 were shown to rapidly and reversibly activate dihydrogen, determined *via para*-hydrogen studies and H_2_/D_2_ exchange studies. The ability to activate dihydrogen was exploited in the successful catalytic H/D exchange between H_2_ and D_2_ and the deuteration of 1-hexene. While the exact mechanism is yet to be resolved, the proposed mechanism proceeds *via* formation of a di-deuteride Rh complex, in-line with the *para*-hydrogen studies, and subsequent coordination and deuteride insertion into the alkene.

In 2023, Fässler and co-workers expanded the family of [(Hyp_3_EtGe_9_)M(PPh_3_)] (M = Ni, Pd, Pt) coordinated clusters with a novel synthetic protocol towards the [(Hyp_3_EtGe_9_)Ni(PPh_3_)] (R = Ph, 4-tolyl, ^*i*^Pr, Me) clusters (8–11) (Fig. 3a).^58–60^ The Ni complexes were targeted as 8 was previously reported to possess dynamic behaviour in solution, potentially of interest in catalytic processes. These clusters are accessible through the reaction of Hyp_3_EtGe_9_ with Ni(COD)_2_ and the respective phosphine. Single-crystal X-ray diffractometry (SC-XRD) and X-ray photoelectron spectroscopy studies confirmed that compounds 8–11 exhibit a 10-atom *closo-*[Ge_9_Ni] core where the Ni(0) is coordinated on the surface of the Ge_9_-cluster. These clusters were initially investigated in alkene isomerisation chemistry, and found to isomerise 1-hexene to 2-*E*- and 2-*Z*-hexene. Successful isomerisation of allylcyclohexane, allylbenzene and COD was also achieved, Fig. 3b. A trend in catalytic performance can be observed where clusters 8 and 9 outperform clusters 10 and 11, with 11 having significantly worse performance. This discrepancy originates from the different coordinating phosphine ligands. During the catalysis performed with 8–10 uncoordinated phosphine can be observed whereas no PMe_3_ was observed when 11 was employed. This indicates a necessity for the phosphine ligand to dissociate to generate a vacant Ni coordination site. Experimental and computational studies indeed indicated that 8 undergoes phosphine liberation and subsequent ligand exchange reactions with toluene solvent to form [(Hyp_3_EtGe_9_)Ni(η^2^-C_6_H_5_CH_3_)], see Fig. 3c. The toluene adduct is subsequently substituted by the 1-alkene and isomerisation to 2-alkene at the nickel centre occurs in an almost thermoneutral step.

**Fig. 3:**
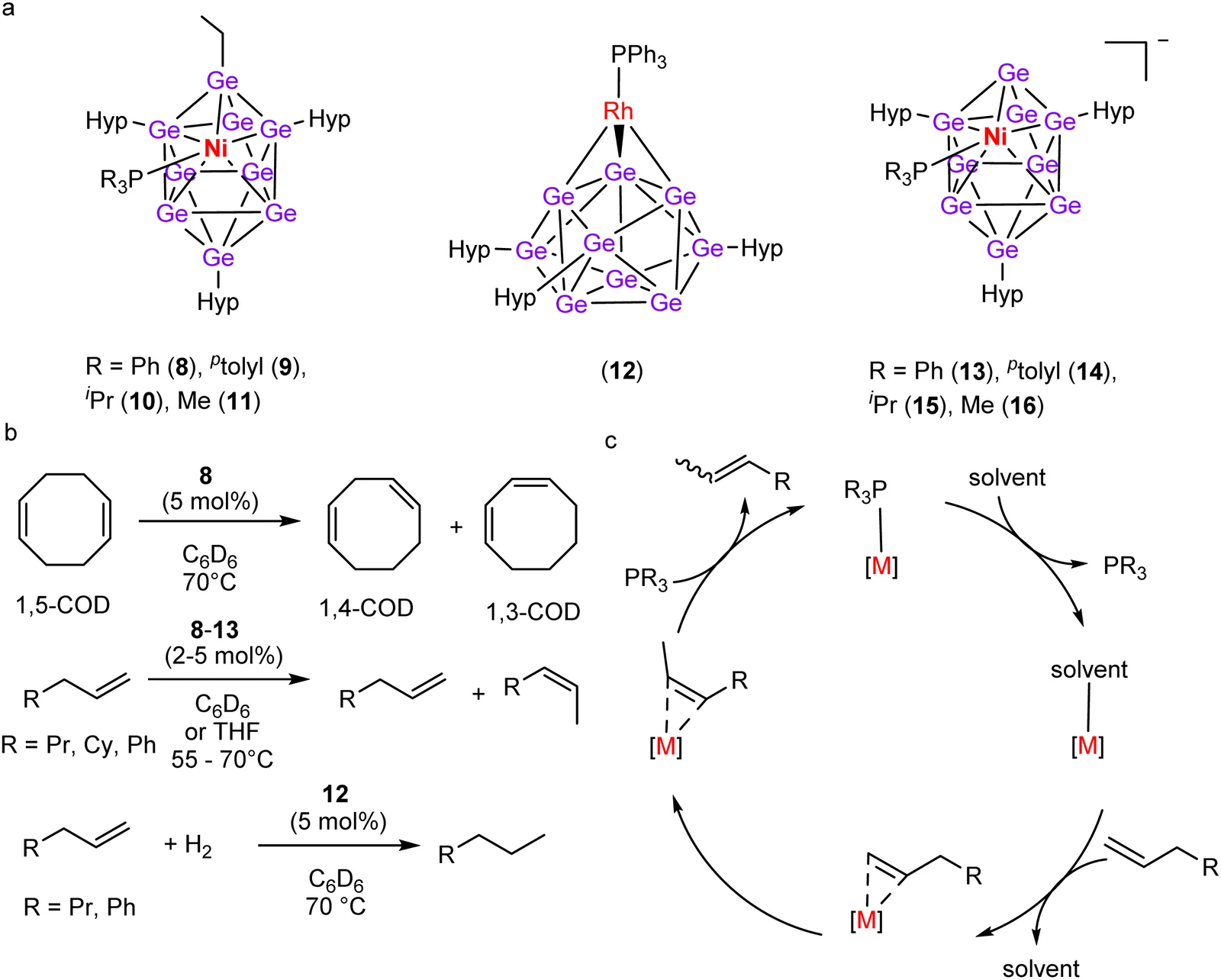
(a) Catalysts 8–16. (b) Catalytic isomerisation and hydrogenation of alkenes. (c) Mechanism of alkene isomerisation.

These transformations were also possible using the related [(Hyp_3_Ge_9_)Rh(PPh_3_)] cluster (12) reported by Fässler and co-workers in 2024.^61^ Compound 12 is readily accessible from K[Hyp_3_Ge_9_] and Rh(PPh_3_)_3_Cl. Like the Ni analogue, compound 8, compound 12 readily isomerises 1-hexene to 2-*E*-hexene and 2-*Z*-hexene, however unlike 8, isomerisation of allylbenzene only resulted in 11% conversion to the 2-*E*- and 2-*Z*-isomers. The difference between 8 and 12 is believed to be due to the cleavability of the phosphine ligands and the presence of excess PPh_3_ co-crystallised with 12. Under the same conditions the hydrogenation of 1-hexene and allylbenzene was also trialled using 1 bar of H_2_. Successful hydrogenation of 1-hexene to hexane and allylbenzene to 1-phenylpropane was observed in 64% and 47% conversion, respectively. A small amount of isomerisation occurred as a competitive reaction under these reaction conditions.

Structurally related to their recently developed [(Hyp_3_EtGe_9_)Ni(PPh_3_)] (R = Ph, 4-tolyl, ^*i*^Pr, Me) clusters (8–11), in 2025 Fässler and co-workers reported the charged analogues [(Hyp_3_Ge_9_)Ni(PR_3_)]^−^ (R = Ph, 4-tolyl, ^*i*^Pr, Me) (13–16) (Fig. 3a).^62^ Due to the charged nature of these [(Hyp_3_Ge_9_)Ni(PR_3_)]^−^ clusters, a dynamic process involving the cluster atoms where the Ni atoms alternate between η^5^- and η^3^-coordination is observed by NMR spectroscopy. A similar dynamic process has been reported by Goicoechea, Weller and co-workers for the [(Hyp_3_Ge_9_)Rh(dppe)] cluster. Compound 13 was tested in the isomerisation of 1-hexene and under the optimised reaction conditions (*i.e.*, 5 mol% loading at 70 °C in tetrahydrofuran (THF)) a conversion of 88% to 2-*E*- and 2-*Z*-hexene was observed (Fig. 3b). Solvent was found to have a significant influence on the overall conversions observed, with THF showing 88% conversion and MeCN giving 48% conversion. This solvent effect was in line with exchange of the phosphine by a solvent molecule being favoured, in the case of donor solvents, over the formation of a vacant binding site, resulting in overall reduced catalytic reactivity (Fig. 3c).

### Zintl materials in heterogeneous catalysis

In 2020, Sun and Zhang employed an endohedral [Sn_9_]^4−^ Zintl cluster [Ru@Sn_9_]^6−^ (17) as a molecular precursor to develop a heterogeneous catalyst for the reverse-water-gas-shift reaction (Fig. 4).^63^ Specifically, the [Ru@Sn_9_]^6−^ cluster was synthesised as previously described by Fässler and co-workers *via* the high temperature reaction of Sn, Ru, and K followed by extraction with ethylenediamine and pyridine.^64^ Then, this cluster was dispersed onto CeO_2_ particles *via* an incipient wetness impregnation method. Energy-dispersive X-ray spectroscopy confirmed homogeneous distribution of K, Ru, and Sn on the CeO_2_ particles, and X-ray photoelectron spectroscopy revealed partial oxidation of the Sn to SnO_2_ and SnO. The Ru-SnO_*x*_/CeO_2_ material obtained was subsequently used in CO_2_ hydrogenation and showed 95% selectivity towards CO formation. This reactivity could be influenced very efficiently by washing with water and altering the water pressure in the system, with a switch to 100% methanation upon >15 kPa water pressure. While the exact structure of the functionalised Ru-SnO_*x*_/CeO_2_ material remains unknown, the use of Zintl clusters as precursors towards catalytically active heterogeneous materials was established in this work.

**Fig. 4:**
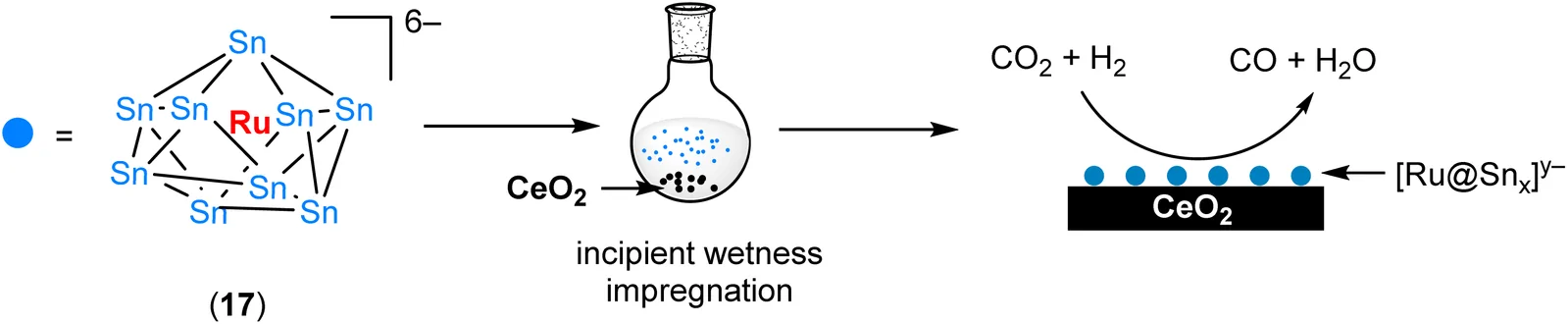
Catalytic reverse-water-gas-shift reaction mediated by Ru-SnO_*x*_/CeO_2_.

In 2019 and 2021, Goldberger reported further developments in Zintl phases as heterogeneous catalysts, this time focused on alkyne hydrogenation chemistry.^65–67^ This work resembles reported heterogeneous catalysis by intermetallic materials, including intermetallic Ga compounds, reviewed previously.^68^ Inspired by bulk hydride materials, Goldberger targeted Zintl phases including those featuring s-, d-, and f-block metals, namely BaGa_2_, LaGa_2_, YGa_2_, BaInGe, *o*-EuGa_2_, h-EuGa_2_, CaIn_2_, CaGaSi, 4*H*-CaGaGe (4-layer hexagonal), and 13T-CaGaGe (13-layer polytype). These Zintl phases display layered structures built from honeycomb layers of Group 13 (Ga, In) or Group 14 (Si, Ge) layers separated by electropositive cations (Ca, Ba, Y, La, Eu), the BaGa_2_ phase is given as an example in Fig. 5a. Initially BaGa_2_ was studied, and this phase was described as a hydrogen absorbing layered Zintl phase due to its Ga atoms featuring a half-filled p-orbital and subsequent propensity to form hydrides. BaGa_2_ was shown to react with dihydrogen to form the bulk gallium-hydride phase, BaGa_2_H_2_, whilst retaining a honeycomb structure now displaying puckering due to sp^3^-hybridised Ga. BaGa_2_ was found to be a remarkably potent catalyst in the hydrogenation of phenyl acetylene, Fig. 5b, giving competitive activities to precious metal catalysts, including those featuring Ni, Pd and Au.^69–75^ Hydrogenation surface specific activities of 425 h^−1^ were observed under mild conditions (40 °C, 1 bar H_2_, 4 mol% catalyst loading), while much higher activities (>8390 h^−1^) were observed at elevated H_2_ pressures (51 bar). The authors note that these values represent the lower bounds of turnover frequency, as probably not every surface Ga atom is catalytically active.

**Fig. 5:**
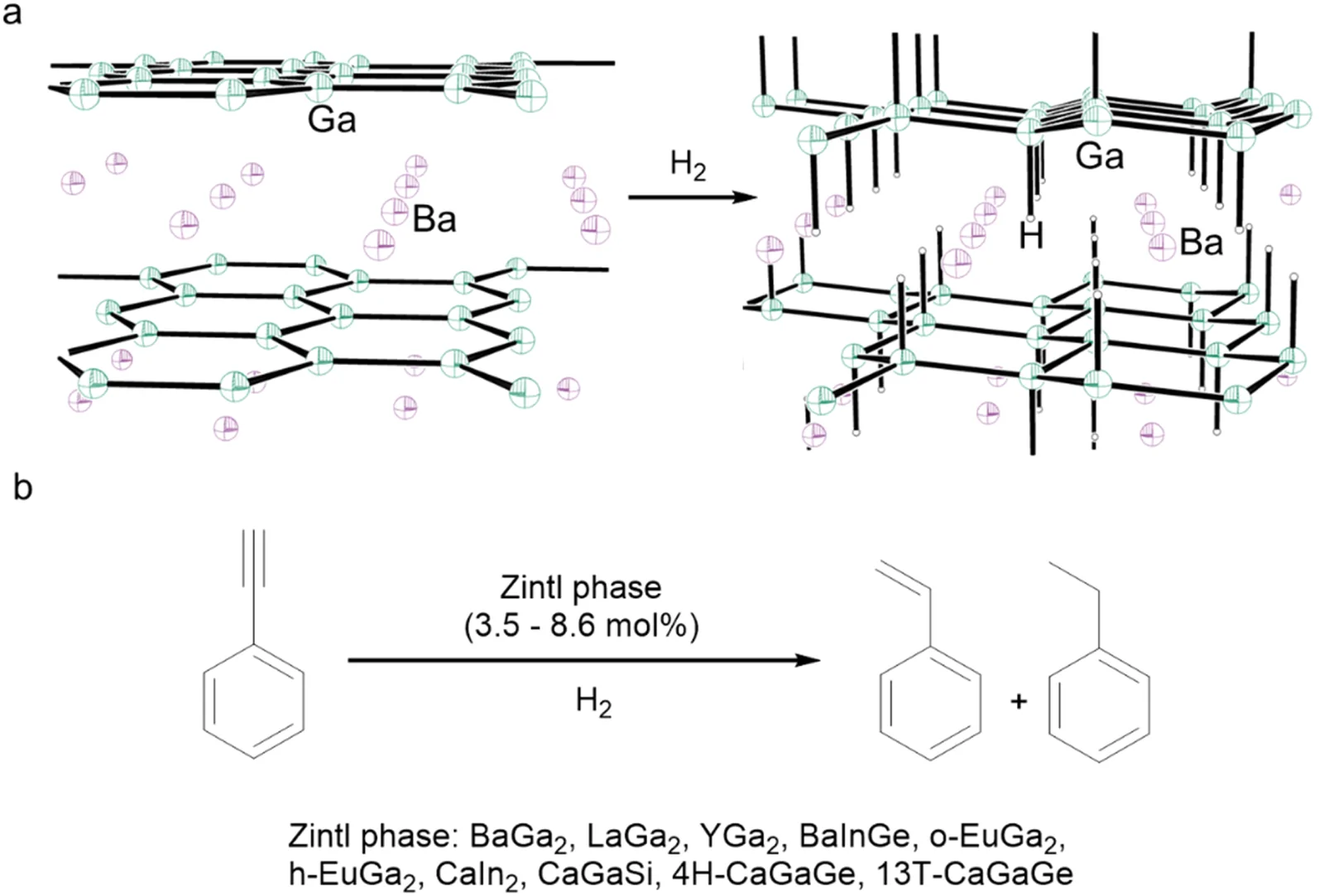
(a) Extended lattice of BaGa_2_ and BaGa_2_H_2_ phases. Drawn from available SC-XRD data.^76,77^ (b) Catalytic hydrogenation of phenyl acetylene by BaGa_2_, LaGa_2_, YGa_2_, BaInGe, *o*-EuGa_2_, h-EuGa_2_, CaIn_2_, CaGaSi, 4H-CaGaGe, and 13T-CaGaGe Zintl phases.

Following on from these studies with BaGa_2_, the isostructural family of phases listed above were tested in the hydrogenation of phenyl acetylene to potentially identify trends as the catalyst is tuned. The selectivity towards styrene or ethylbenzene could be influenced by the choice of solvent, with high selectivity achieved with most phases. *o*-EuGa_2_ and CaIn_2_ with interlayer bonding between the Group 13 elements demonstrated the lowest catalytic activity, presumably due to the lack of half-filled p-orbitals. This was neatly shown by the h-EuGa_2_ phase which was found to be much more catalytically active than *o*-EuGa_2_ while containing the same elements but no interlayer bonding. LaGa_2_ was found to be unreactive but this was presumed to be due to its extreme air sensitivity and subsequent decomposition. All other phases showed appreciable conversion with BaInGe being outperformed by all Ga-containing phases, indicating higher activity of Ga over In. CaGaSi, YGa_2_, BaGa_2_, and 13T-CaGaGe were found to be the most active. These phases have different total valence electrons per formula unit and are either planar or distorted, indicating that high catalytic performance can be achieved when either one or both of the main group elements on the framework feature a half-filled p-orbital and that catalytic activity does not depend on small distortions away from planarity. The 13-layer polytype 13T-CaGaGe phase was found to be the most active with a surface specific activity of 590 h^−1^ at 1 bar H_2_. Remarkably, upon exposure to air the 13T-CaGaGe phase did lose some catalytic competency; however, it remained highly active in high pressure reactions even when the material was exposed to air for over 5 months. Further recycling experiments revealed excellent recyclability of the 13T-CaGaGe phase. Overall, this work elegantly shows the potential of heterogeneous Zintl phases in common transformations such as hydrogenation reactions, which are currently the domain of expensive and scarce precious metals.^78,79^ Furthermore, important work remains to be done to elucidate the nature of catalytically active sites in processes mediated by heterogeneous Zintl phases and heterogeneous catalysts synthesised from Zintl cluster-based precursors. Establishing whether the active catalysts in these materials retain the initial Zintl motif, or how this motif is altered, will be imperative for the rational design and improvement of these catalytically active materials.

### Emerging main group small molecule activations

The blossoming field of transition metal-free Zintl cluster catalysis is dominated by the [Pn_7_] family of clusters (Pn = pnictogen; P, As), the solution-state chemistry of which has been reviewed previously.^13,18^ Briefly, [Pn_7_] clusters exhibit some remarkable bonding and structural aspects that are foundational to their chemistry. Unlike tetrel-derived Zintl clusters, *e.g.* [Ge_9_]^4−^, which commonly exhibit delocalised bonding and are often discussed using Wade–Mingos rules, [Pn_7_] Zintl clusters exclusively contain two-centre two-electron bonding. They feature three basal P atoms connected to an apical P centre *via* three bridging P centres, which in the case of the naked trianionic salt can be considered phosphide-like with a formal oxidation state of −1. These anionic bridging sites are potent bases and are strongly reducing.^18^ The seminal and pioneering work by Baudler and Eichhorn towards the characterisation and functionalisation of [Pn_7_] clusters have been previously reviewed and highlighted.^80–82^ Here, we discuss only recent reports of stoichiometric small molecule activations with (functionalised) Zintl clusters that laid the groundwork for the emerging field of transition metal-free Zintl cluster catalysis.

Tetrel-functionalised [P_7_] and [As_7_] Zintl clusters have been explored in small molecule activation in a manner that is reminiscent of the chemistry of frustrated Lewis pairs (FLPs).^83,84^ Heteroallenes, namely isocyanates (RN=C=O) and isothiocyanates (RN=C=S), were found to insert into each of the three P–Si bonds present in the silyl-functionalised Zintl cluster, TMS_3_P_7_ (TMS = trimethylsilyl) (18) (Fig. 6).^85^ This insertion chemistry is akin to the hydrophosphination chemistry of [HP_7_]^2−^ with heteroallenes previously reported by Goicoechea and co-workers.^86–88^ Treatment of 18 with three equivalents of phenyl isocyanate afforded compound (19) in which each of the bridging phosphorus atoms of the [P_7_] core binds to an isocyanate C atom, two of the TMS groups bind to isocyanate nitrogen atoms and one TMS group binds to an isocyanate O atom. As such, compound 19 features one imidate group [RC(=NR)OR] and two amide groups [RC(=O)NR] (an “*N*,*N*,*O*-coordination mode”), as confirmed by SC-XRD and ^29^Si NMR studies (Fig. 6).

**Fig. 6:**
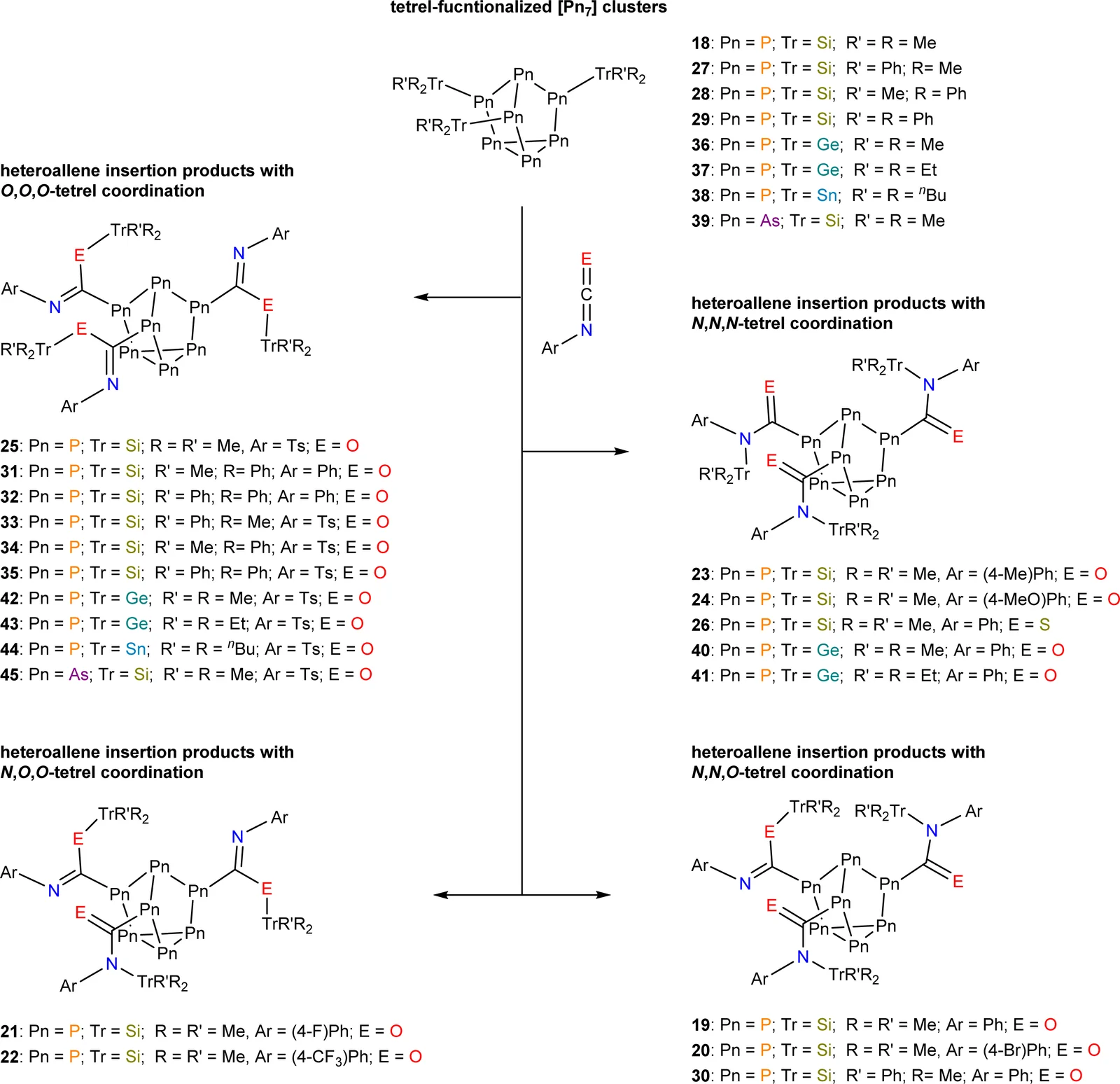
Synthesis of 18–45*via* heteroallene insertion into tetrel-functionalised [Pn_7_] clusters.

Similar reactivity was observed when 18 was treated with aryl isocyanates bearing electron-withdrawing or donating groups; however, the position of the TMS groups on the resulting compounds varied (Fig. 6).^85^ Treatment of 18 with 4-bromophenyl isocyanate, 4-fluorophenyl isocyanate, 4-trifluoromethylphenyl isocyanate, 4-tolyl isocyanate, 4-methoxyphenyl isocyanate, or 4-tosyl isocyanate afforded compounds 20–25, respectively. Compound 20 features one imidate group and two amide groups, compounds 21 and 22 feature two imidate groups and one amide group (an “*N*,*O*,*O*-coordination mode”), compounds 23 and 24 feature three amide groups (an “*N*,*N*,*N*-coordination mode”), and compound 25 features three imidate groups (an “*O*,*O*,*O*-coordination mode”). Further, treatment of 18 with phenyl isothiocyanate affords 26, which features three N–Si bonds. With these data in hand, it could be concluded that introduction of electron-withdrawing groups to the isocyanate aromatic ring results in preferential TMS coordination to the isocyanate O atom over the N atom. It is also worth noting that in this work, there is no evidence for fluxional coordination where the silyl group “hops” between N and O coordination modes.

The authors also isolated silyl-functionalised clusters 27, 28, and 29 to elucidate the effect of varied silyl groups on these isocyanate insertion reactions (Fig. 6).^85^ Treatment of 27, 28, and 29 with either phenyl or 4-tosyl isocyanate afforded 30–35. The authors found that reaction times across the series differed dramatically, with the reaction between 29 and phenyl isocyanate taking up to 30 days to complete. Beyond increased reaction times, the Ph-substituted silyl groups tended to prefer O-coordination over N-coordination; this preference can be attributed to both electronic and steric factors. Remarkably, when compound 26 was treated with aryl isocyanates, the authors observed clean formation of the respective aryl isocyanate insertion products *via* displacement of the captured phenyl isothiocyanate, reminiscent of reversible FLP small molecule binding. While insertion of heteroallenes into P–Si bonds with simple silylphosphines has been reported, this ability to exchange the inserted heteroallene is not reported with these simple silylphosphines.^89,90^ The ability for substrates to reversibly insert into the P–Si in these clusters is compelling for the further development of tetrel-functionalised [Pn_7_] clusters in catalysis; such species could complement the reactivity of more developed triel-functionalised Zintl cluster platforms (*vide infra*).^91^

In a subsequent report, compounds (Me_3_Ge)_3_P_7_ (36), (Et_3_Ge)_3_P_7_ (37), (^*n*^Bu_3_Sn)_3_P_7_ (38), and (TMS)_3_As_7_ (39) were treated with phenyl isocyanate, 4-tosyl isocyanate, and phenyl isothiocyanate (Fig. 6).^92^ Compounds 36 and 37 reacted with phenyl isocyanate to afford compounds 40 and 41. It is hypothesised that the lower oxophilicity of Ge centres relative to Si centres results in N-coordination as opposed to O-coordination in 40 and 41. Compounds 38 and 39 did not react with phenyl isocyanate. Compounds 36–39 each react with 4-tosyl isocyanate to form insertion products 42–45, respectively. However, compounds 42–45 exhibit O-coordination, presumably due to the lower basicity of the isocyanate N atom. Compounds 36–39 were not found to react with phenyl isothiocyanate.

Compound 18 could be treated with 1 atm CO_2_ in the presence of THF to cleanly afford the CO_2_ insertion product 46 (Fig. 7).^92^ Ultimately, the activation of heteroallenes, and the lability of the resulting heteroallene insertion products marked a clear advancement towards transition metal-free substrate activation at Lewis acid-functionalised Zintl clusters.

**Fig. 7:**
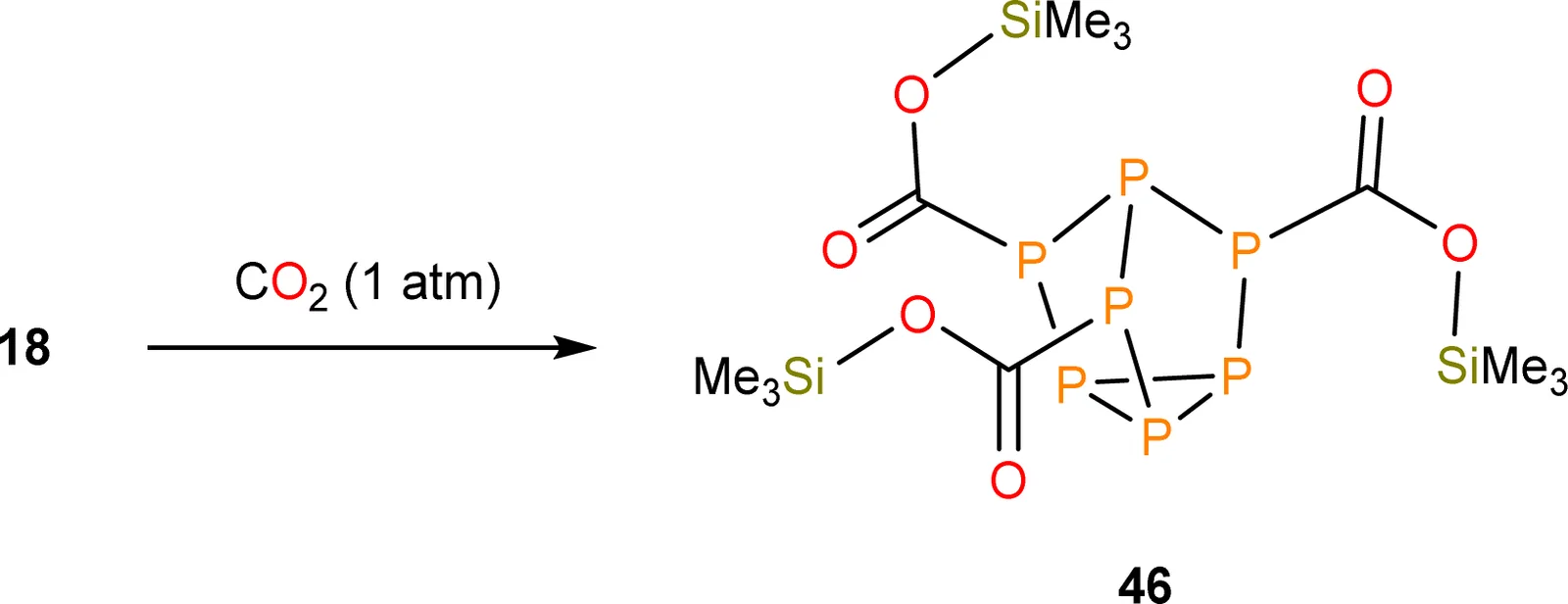
Synthesis of 46.

The activation of heteroallenes by tetrel-functionalised [P_7_] clusters was extended to azides (Fig. 8).^93^ When compounds 18 or 36 were reacted with benzyl azide, an immediate colour change accompanied by effervescence was observed, suggesting the expected insertion reactions had occurred accompanied by the loss of N_2_. In line with these observations, compounds 18 and 36 had been consumed during the course of the reaction to form the tris-insertion products 47 and 48, respectively, as confirmed by ^31^P NMR and SC-XRD studies.

**Fig. 8:**
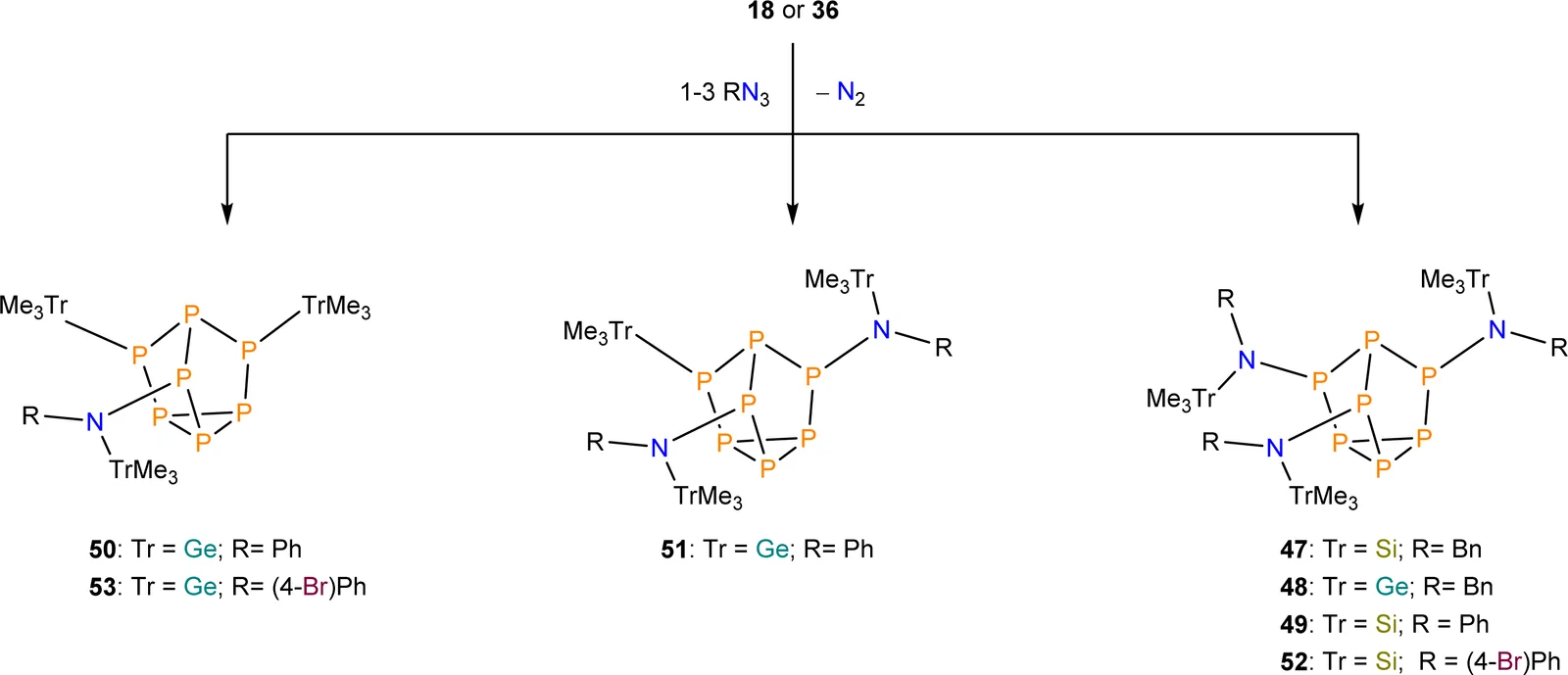
Synthesis of 47–53*via* azide insertion into 18 or 36.

Compound 18 was treated with phenyl azide to afford the related tris-insertion product 49; however, treatment of compound 36 with phenyl azide afforded a mixture of mono- and bis-inserted products (50 and 51, respectively) which were detected by ^31^P NMR spectroscopy and mass spectrometry (Fig. 8).^93^ Furthermore, the mono-inserted product was structurally characterised. Treatment of compound 18 with 3 equivalents of 4-bromophenyl azide readily afforded the expected tris-inserted product 52. In contrast, treatment of compound 31 with 3 equivalents of 4-bromophenyl azide yielded only trace amounts of the tris-inserted product in a mixture that also contained the mono- and bis-inserted products in addition to cluster decomposition products. Treatment of 36 with 1 equivalent of 4-bromophenylazide resulted in the clean formation of the mono-inserted product 53 as the major product. The observation of varied mono-, bis-, and tris- insertion products from the reactions with azides contrasts the analogous reactions with isocyanates, which only generated tris-insertion products.

Besides Group 15 clusters, the anionic Group 14 cluster [Hyp_2_Ge_9_]^2−^ has also been investigated in the context of metal-free small molecule activation.^94^ Reaction of [Hyp_2_Ge_9_]^2−^ with DAB^*o*-tol^–Br (DAB = 1,3,2-diazaborolidine; 2-tol = 2-methylphenyl) in dioxane resulted in the formation of the borane-functionalised cluster, [(Hyp_2_Ge_9_)(DAB^*o*-tol^)]^−^, while the same reaction in the presence of THF afforded the ring opened product, [(Hyp_2_Ge_9_){(CH_2_)_4_O}(DAB^*o*-tol^)]^−^ (Fig. 9). When the more sterically encumbered borane, DAB^Mes^–Br (Mes = 2,4,6-trimethylphenyl), was employed, no adduct formation between [Hyp_2_Ge_9_]^2−^ and the borane is observed, and the mixture of the two species in the presence of either THF or trimethylene oxide (TMO) leads to the ring-opened products, [(Hyp_2_Ge_9_){(CH_2_)_4_O}(DAB^Mes^)]^−^ (54) or [(Hyp_2_Ge_9_){(CH_2_)_3_O}(DAB^Mes^)]^−^ (55), respectively. Moreover, treatment of a mixture of [Hyp_2_Ge_9_]^2−^ and DAB^Mes^–Br with MeCN led to the nitrile-activated product, [(Hyp_2_Ge_9_){MeCN}(DAB^Mes^)]^−^ (56), *via* a FLP-type mechanism in which the [Ge_9_] unit acts as the Lewis base and DAB^Mes^ acts as the Lewis acid (Fig. 9). This nitrile-activated product has subsequently been studied by optical spectroscopy and time-dependent DFT to show charge-transfer processes between the cluster and activated nitrile fragment, which could be an interesting starting point for substrate photoactivation in new bond-breaking or bond-forming processes.^95^ However, thus far, functionalised [Ge_9_] clusters have yet to be explored in the context of transition metal-free catalysis.

**Fig. 9:**
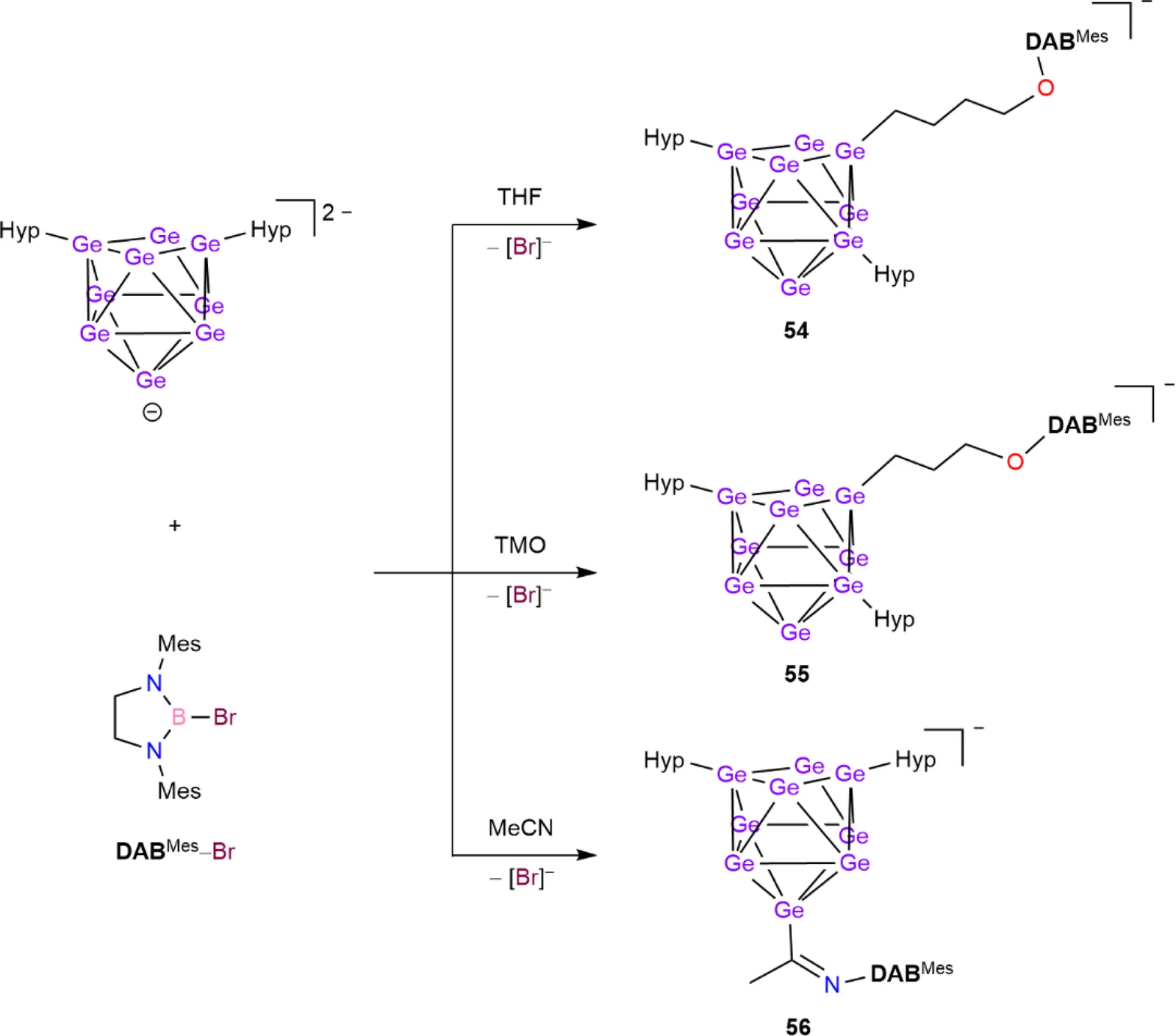
Reactions between a mixture of [Hyp_2_Ge_9_]^2−^ and DAB^Mes^-Br with either THF, TMO, or MeCN to afford 54, 55, or 56, respectively. DAB = 1,3,2-diazaborolidine.

### Zintl clusters in transition metal-free catalysis

#### Zintl cluster as an innocent platform

Zintl clusters have served as an innocent platform upon which boron-centred reactivity could be mapped. [Na(DME)_*x*_]_3_[P_7_] (DME = dimethoxyethane) was treated with an excess of chlorodimethylvinylsilane to afford compound 57, which features three P–Si bonds that covalently tether the olefin functional motifs to the [P_7_] platform (Fig. 10a).^96^ Subsequent treatment of 57 with 1.5 equivalents of 9-borabicyclo(3.3.1)nonane dimer (HBBN dimer) affords the desired borane-tethered cluster, 58. Compound 58 could similarly be prepared by treatment of [Na(DME)_*x*_]_3_[P_7_] with an excess of the pre-formed borane tether, ClSiMe_2_CH_2_CH_2_–BBN. The Lewis acidity of 58 was probed with the Gutmann–Beckett test and fluoride ion affinity (FIA) calculations, and the results obtained were comparable to those found for the free borane ClSiMe_2_CH_2_CH_2_–BBN indicating that tethering to the [P_7_] platform should not attenuate the Lewis acidity and catalytic potential of the boron centre, which was experimentally validated.

**Fig. 10:**
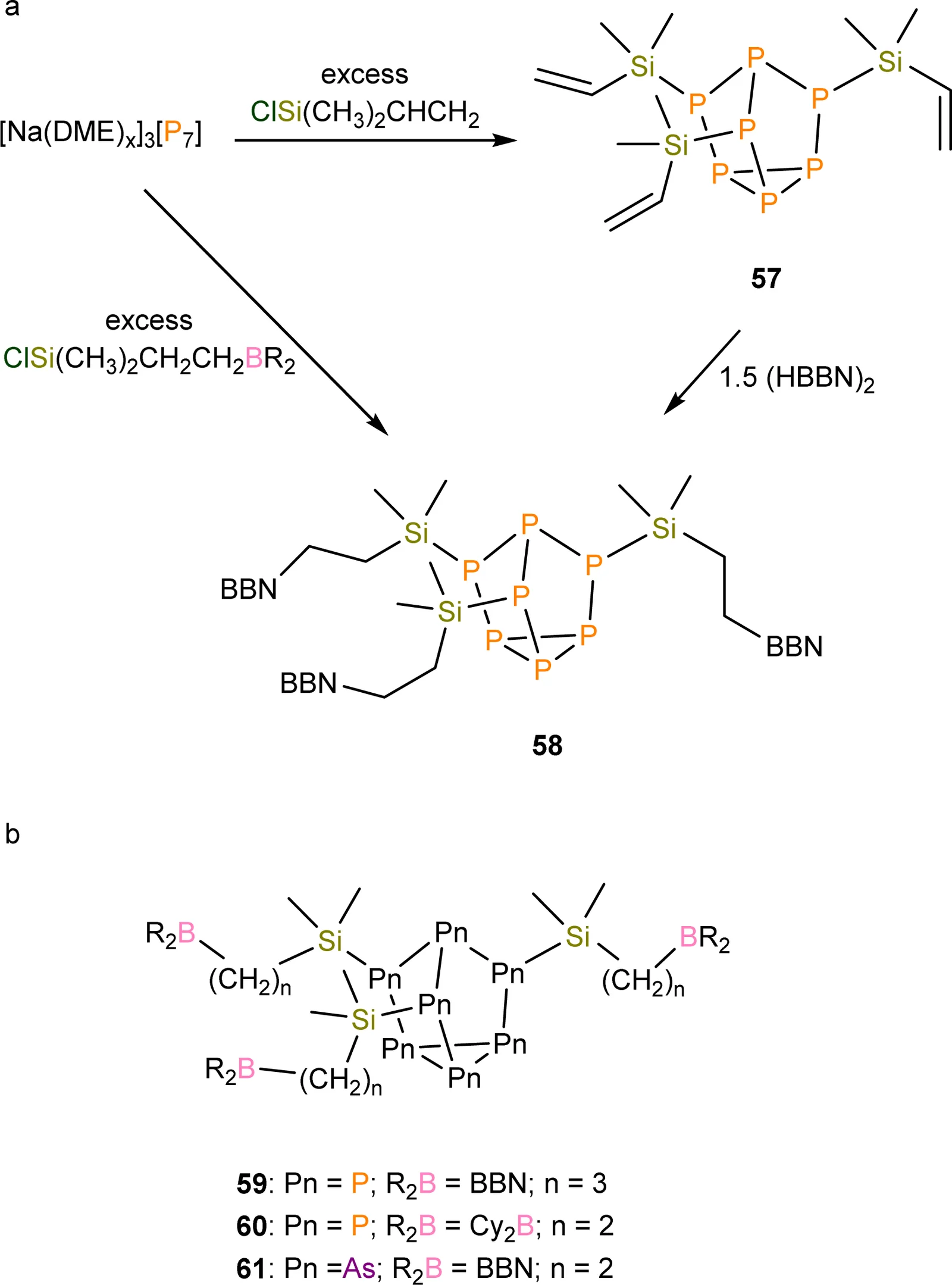
(a) Synthesis of 57 and 58. (b) Catalysts 59–61.

The hydroboration of unsaturated organic functional groups is crucial in the production of many commodity chemicals, and Lewis acidic boranes often exhibit high catalytic efficiencies in these synthetic processes.^97^ Compound 58 was found to catalyse hydroboration reactions between pinacol borane (HBpin) and a range of unsaturated substrates, including carbodiimides, isocyanates, ketones, alkenes, alkynes, and nitriles, with excellent efficiency.^96^ Mechanistic studies confirmed that the catalyst 58 was not degraded and consumed during these transformations. As such, this work represents the first demonstration of Zintl-based clusters as an innocent platform in metal-free catalysis. More broadly, this work demonstrates that a desirable, reactive boron-site can be mapped onto an innocent Zintl cluster platform, which could lead to applications of these clusters in other classical borane chemistry, *e.g.*, neutron capture therapy, cross-coupling reactions to build polymers, and FLP chemistry.^83,97,98^ The authors highlight that functionalised Zintl clusters are reminiscent of boron-functionalised polymers, which are currently under intense investigation as stimuli-responsive, optical, self-healing, and sensor materials.^99,100^

In subsequent work, the authors capitalised on the modular design of catalyst 58 to develop a library of related borane-tethered Zintl clusters.^101^ Following similar synthetic methodologies, compounds 59–61 were developed, where the organic linker, the [Pn_7_] cluster, and the identity of the tethered borane were altered (Fig. 10b).^101^ Compound 59 features three BBN units tethered to a [P_7_] cluster by a longer C_3_ (–CH_2_CH_2_CH_2_–) motif relative to 58. Compound 60 is identical to 58, but the BBN units are replaced by BCy_2_ units, while compound 61 is identical to 58, but features a [As_7_] core rather than a [P_7_] unit. The Lewis acidity of catalysts 59–61 was probed experimentally by the Gutmann–Beckett method and computationally by comparing FIA and hydride ion affinities (HIA) values. Catalytic performance was compared in model Lewis acid-catalysed hydroboration reactions. It was found that the Lewis acidity, and by proxy the catalytic performance of these clusters was almost entirely dependent on the identity of the tethered borane, and the free boranes performed similarly to their tethered counterparts. These findings ultimately suggested that the Zintl cluster can indeed act as an innocent platform for boron-centred Lewis acid catalysis, and that the tethering process does not perturb the chemistry of these mounted reactive sites. More broadly, the ability to tether Zintl ions *via* organosilanes to alternate chemical units may prove useful in the incorporation of Zintl clusters into varied functional materials.

### Zintl clusters as a non-innocent platform for catalysis

Zintl clusters are now known to play an active role in catalysis through diverse functions including cluster-centered Lewis basicity and redox cycling. Inspired by the success of FLP chemistry, the Mehta group designed the first crystallographically characterised boron-functionalised heptaphosphide Zintl cluster with the boron moiety directly bonded to the cluster, [Na(18c6)]_2_[(BBN)P_7_] (62). Compounds 62 and 63 were expected to cooperatively activate substrates between the Lewis basic phosphide sites within the [P_7_] unit and the Lewis acidic boron centre, which could be exploited in catalysis (Fig. 11).^91^ Compound 62 was synthesised *via* a dehydrocoupling reaction between [Na(18c6)]_2_[HP_7_] and HBBN dimer, and [K(18c6)]_2_[(BBN)P_7_] (63) was prepared similarly.

**Fig. 11:**
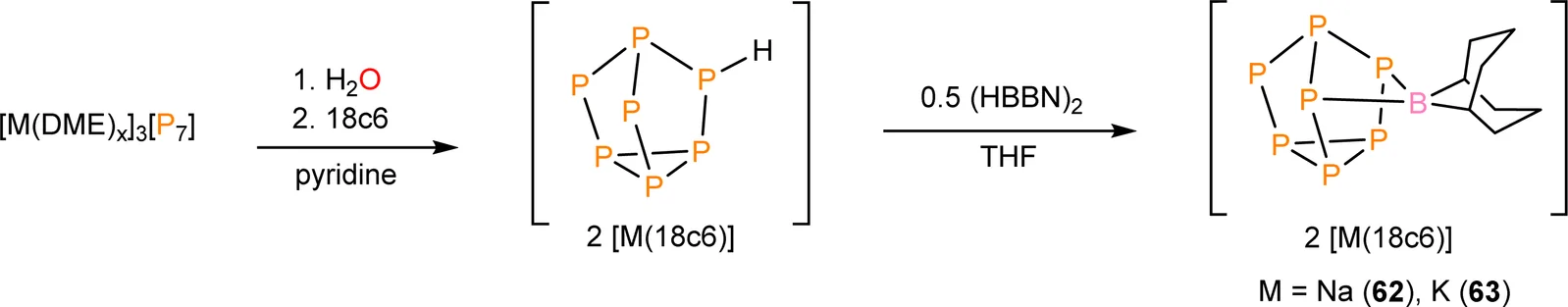
Synthesis of catalysts 62 and 63.

The novel Lewis-acid functionalised cluster compounds, 62 and 63, were initially tested in the reduction of carbonyl groups.^91^ Remarkably, 62 and 63 each achieved >99% conversion of benzaldehyde or benzophenone to afford the respective benzyloxyboranes at room temperature in *ortho*-difluorobenzene (oDFB) with 1 mol% catalyst loading (Fig. 12a). Under these optimised conditions, the authors showed efficient hydroborative reduction of aldehydes and ketones, as well as heteroallenes, including isocyanates and carbodiimides (Fig. 12b). Most notably, the authors found that compound 62 efficiently catalyses the conversion of CO_2_ and HBBN dimer to MeOBBN under mild conditions (Fig. 12c), achieving excellent turnover numbers (TON) and turnover frequencies (TOF) relative to other main group catalysts (up to TON = 9800 at 0.01 mol% and TOF = 300 at 0.33 mol%).^91^

**Fig. 12:**
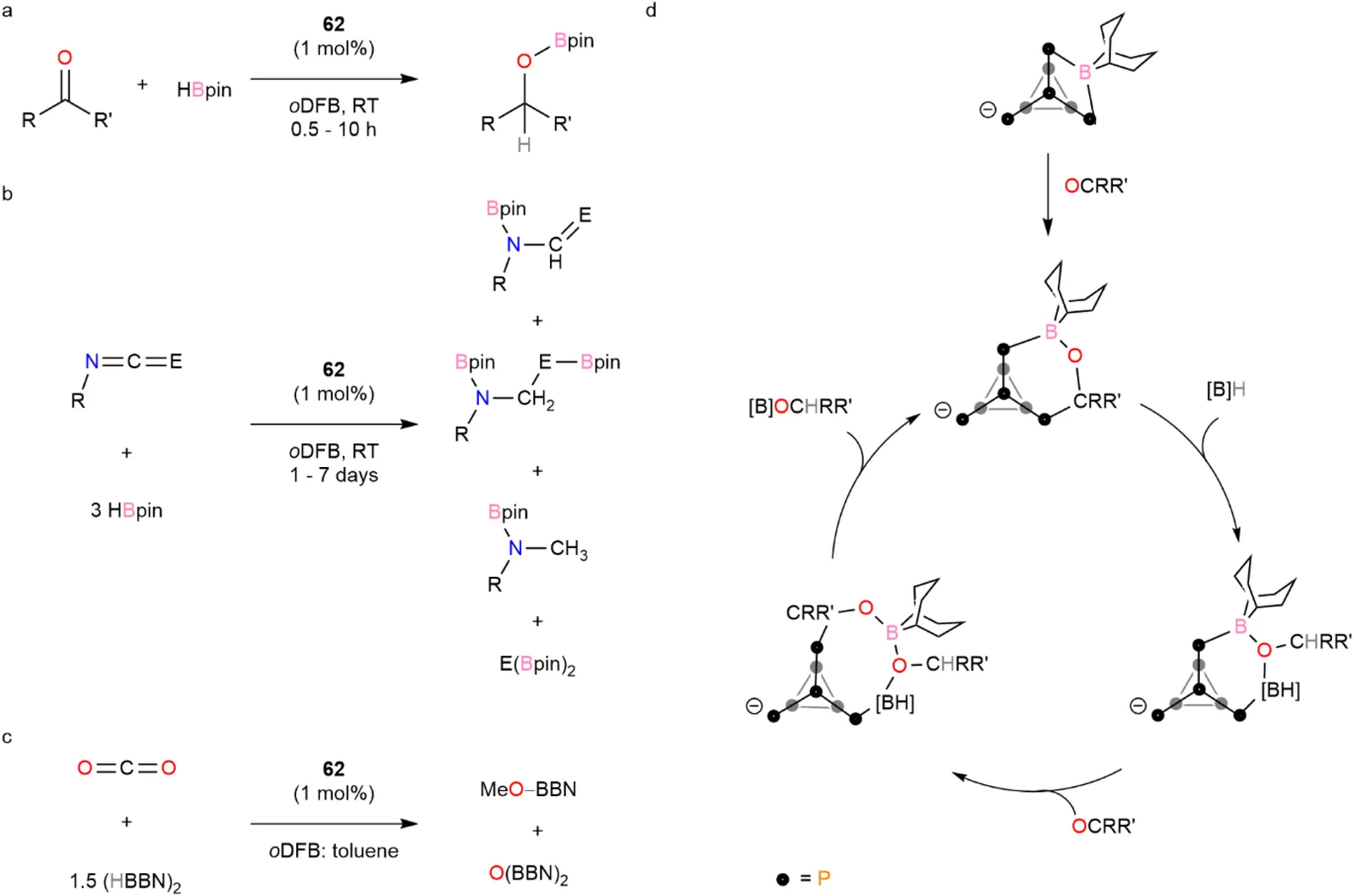
Catalytic hydroboration (a) aldehydes and ketones, (b) isocyanates and carbodiimides, and (c) CO_2_ with 62. (d) Proposed mechanism for the catalytic hydroboration of carbonyls by 62 ([B]H = borane reductant).

Computational and experimental mechanistic investigations provide strong support for cooperative reactivity between the Lewis basic phosphorus site and the Lewis acidic boron site (Fig. 12d). The authors propose a catalytic cycle involving an initial carbonyl insertion into the relatively labile P–B bond. The resulting activated carbonyl group is then susceptible to the addition of the B–H bond of the reductant. A second equivalent of carbonyl species is inserted which triggers the elimination of the hydroborylated product. Based on the proposed mechanism, compound 62 is more accurately described as a pre-catalyst for the hydroboration of CO_2_. A recent theoretical investigation was conducted to probe the fluxionality of the anion, [(BBN)P_7_]^2−^, which has been suggested to be an essential feature in the catalytic mechanism of the aforementioned hydroborations.^102^ Notably, this investigation of the free energy surface of [(BBN)P_7_]^2−^ identified two pathways in which the BBN unit can “walk” along the surface of the cluster, making and breaking P–B bonds and scrambling the phosphorus sites.^102^

The modular design of compound 62 allows for facile manipulation of the Lewis acidic moiety, the [Pn_7_] cluster core, and the counter cation to elucidate the impact of these components on catalytic performance. Compounds [Na(18c6)]_2_[(^*i*^Bu_2_Al)P_7_] (64) and [K(18c6)]_2_[(^*i*^Bu_2_Al)As_7_] (65) could be prepared as analytically pure materials *via* a dehydrocoupling reaction between ^*i*^Bu_2_AlH and the protonated clusters [HPn_7_]^2−^ (Fig. 13).^103^ Furthermore, the literature-known cluster [(Ph_2_In)P_7_]^2−^ was prepared as both the sodium salt [Na(18c6)]_2_[(Ph_2_In)P_7_] (66) and the potassium salt [K(18c6)]_2_[(Ph_2_In)P_7_] (67) (Fig. 13).^104^ Compounds 64–67 were studied in the catalytic hydroboration of CO_2_ with HBBN dimer. With respect to TOF, the aluminium-containing compounds 64 and 65 showed enhanced performance relative to 62 and 63, and the indium-containing compounds 66 and 67 showed the lowest performance of the series.^103^ The phosphorus analogue 64 showed superior performance over the arsenic analogue 65. At 0.33 mol% catalyst loading and 50 °C, 64 exhibited a remarkably high TOF of 577 h^−1^ (almost twice as high as that of 62 under similar conditions), albeit with lower selectivity towards the desired MeOBBN (65% MeOBBN conversion and 34% CH_2_(OBBN)_2_ conversion). The selectivity could be recovered by reducing the catalyst loading to 0.1 mol% to achieve >99% conversion to MeOBBN with a TOF of 42 h^−1^ at room temperature. At 0.01 mol% of 64 and 50 °C, a TON of 9600 and TOF of 145 h^−1^ could be achieved over the course of a 66 h experiment with 96% conversion of MeOBBN.^103^

**Fig. 13:**
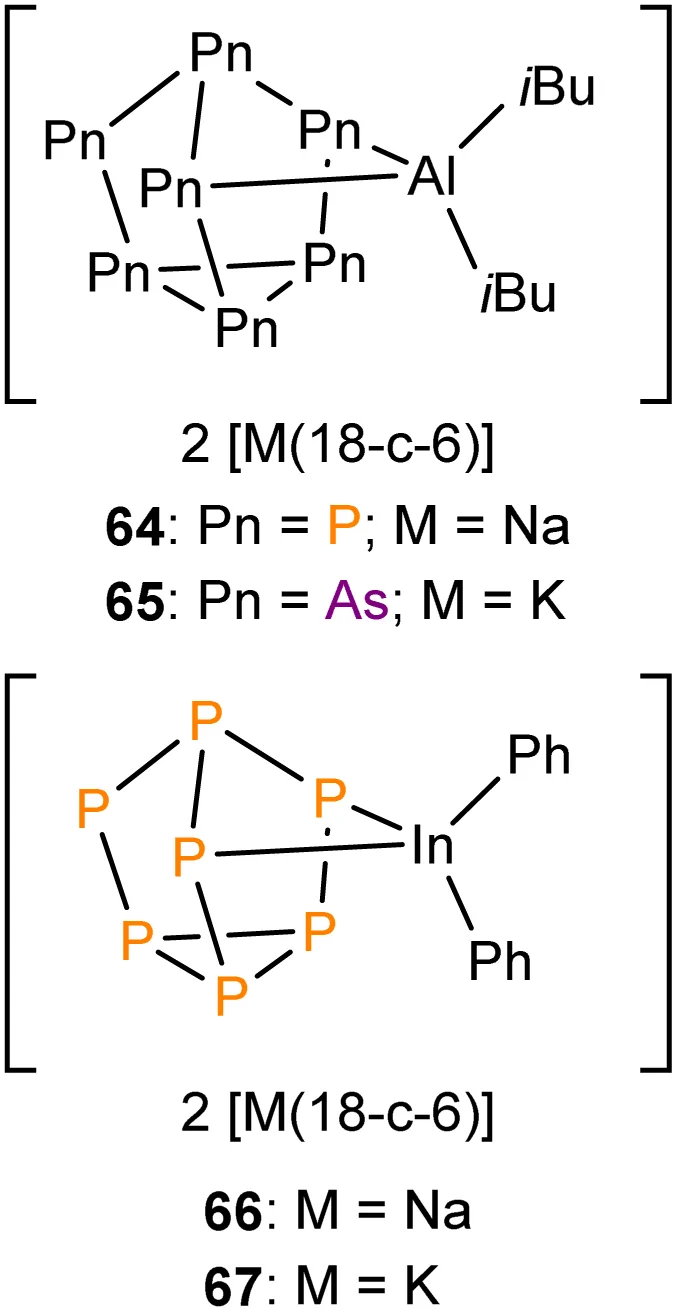
Catalysts 64–67.

Compound 64 outperforms state-of-the-art homogeneous main group catalysts and even numerous transition metal-based systems in this hydroborative CO_2_ reduction. However, it is worth noting that in CO_2_ abatement technology, boranes are not desirable reductants due to their high cost and poor atom economy.^105^ Moreover, although this is high catalytic performance within batch homogeneous catalysis, the TON and TOF values achieved are not yet competitive with state-of-the-art electrocatalytic conditions employed in the field to convert CO_2_ to methanol.^106,107^

Beyond C–O containing substrates, compound 62 was discovered to catalyse the hydroboration of pyridines to dihydropyridine derivatives, an important class of molecules in both nature and medicine.^108^ The hydroboration of pyridine by HBpin was catalysed to >99% conversion with 10 mol% loading of 62 in oDFB at 50 °C (Fig. 14a). Moreover, the reaction was highly selective for the *para*-substituted product over the *ortho*-substituted product, yielding these products in a ratio of 96 : 4, respectively. Compound 63 performed similarly, albeit with significantly lower selectivity, and [Na(DME)_*x*_]_3_[P_7_] only afforded a 70% conversion in the presence of 18c6. Compound 62 could similarly catalyse the hydroboration of various pyridine derivatives, imines, and nitriles (Fig. 14a–c). Experimental and theoretical mechanistic investigations provide support for a mechanism involving an initial heterolytic cleavage of HBpin to install a hydride on the cluster-bound borane and Bpin on a free phosphide site (Fig. 14d). Pyridine then coordinates the P-bound Bpin group and the hydride is then transferred onto the coordinated pyridine, before the hydroborated product is eliminated to regenerate the cluster catalyst. A more in-depth theoretical investigation of the catalytic hydroboration of pyridine by functionalised Zintl clusters was subsequently performed and identified a more complete mechanism.^109^

**Fig. 14:**
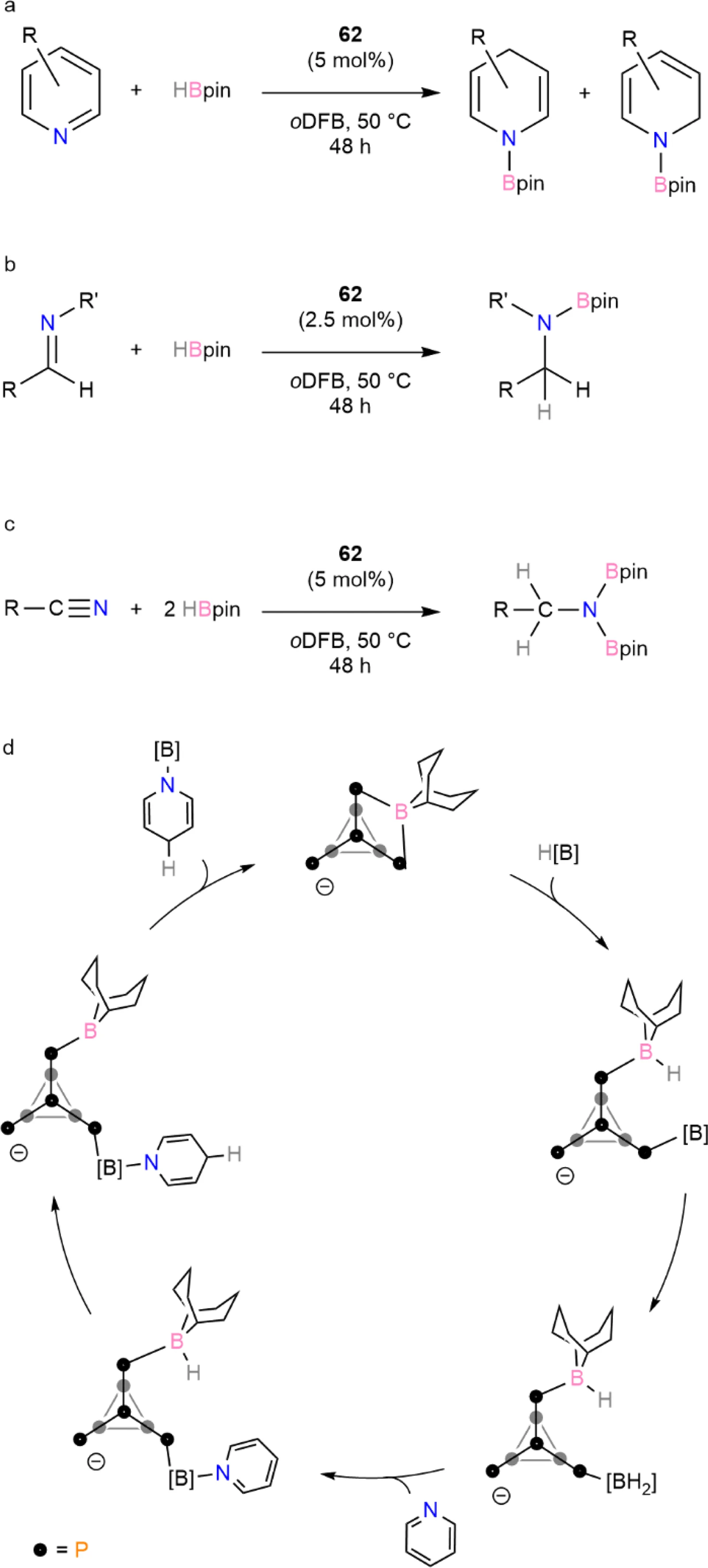
Catalytic reduction of (a) pyridines, (b) nitriles, and (c) imines by 62. (d) Proposed mechanism for the hydroboration of pyridines by 62.

Compound 62 promotes the catalytic hydrophosphination of phenylacetylene with diphenylphosphine in pyridine under mild conditions.^110^ Remarkably, the unfunctionalised Zintl cluster, [Na(DME)_0.05_]_3_[P_7_] (68), was shown to be even more efficient in promoting the hydrophosphination of phenylacetylene (>99% in 24 h), while the reaction did not proceed in the presence of HBBN dimer, compound 18, NaOTf, or 18c6 independently (Fig. 15a). These results demonstrate the role of the cluster-based phosphide sites in establishing catalytic turnover in the reaction. The role of the basic phosphide site is further supported by enhanced efficiency of the hydrophosphination reaction in the presence of cation-sequestering agents 18c6 or 2.2.2.crypt, which disrupt interactions between the Na ions and the phosphide groups. Compound 68 was also found to efficiently promote the hydrophosphination of a range of alkynes, alkenes, and imines at 1 mol% in the presence of 3 mol% 18c6 under mild conditions in pyridine (Fig. 15b–d). Further, a range of Zintl phases, namely, K_3_P_7_, K_3_Sb_7_, K_5_Bi_4_, K_4_Ge_9_, and K_4_Sn_9_ were also found to promote the hydrophosphination of phenylacetylene, with only K_3_Sb_7_ and K_4_Ge_9_ not achieving near quantitative hydrophosphination of phenylacetylene. However, in the presence of 18c6, all Zintl phases studied achieved excellent alkyne hydrophosphination conversions. Mechanistic investigations revealed that the role of the Zintl clusters and phases in this work was as an initiator, which deprotonates diphenylphosphine to form the active catalyst, MPPh_2_ (M = Na, K). Sodium diphenylphosphides are known to catalyse the hydrophosphination of alkynes independently.^111^

**Fig. 15:**
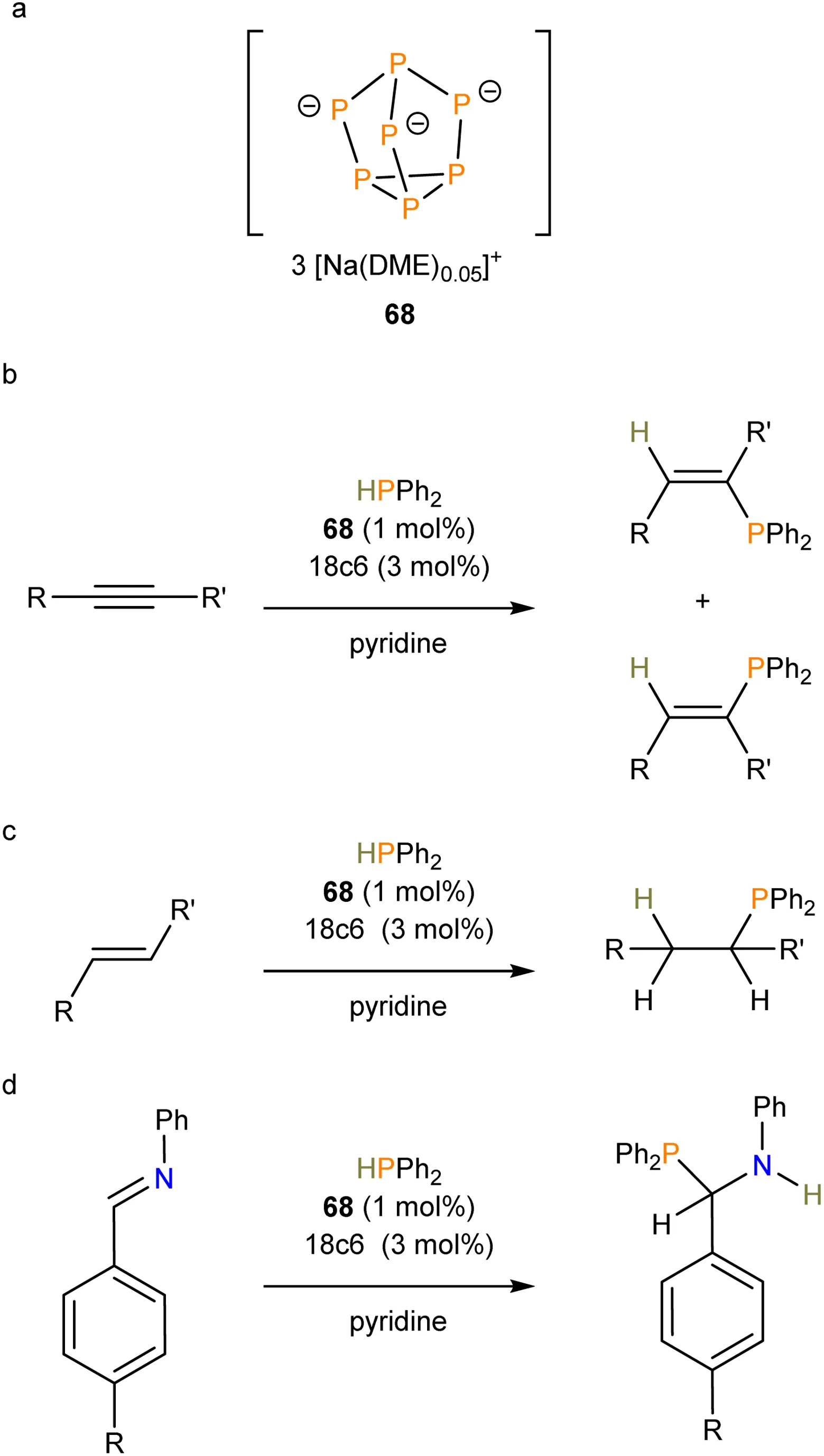
(a) Catalyst 68. Catalytic hydrophosphination of (b) alkynes, (c) alkenes, and (d) imines by 68.

N_2_O is a common industrial pollutant and greenhouse gas with an estimated greenhouse warming potential 300× greater than CO_2_.^112,113^ Compounds 62, 64, 65, 66, [K(18c6)]_3_[P_7_] (69), and [K(18c6)]_3_[As_7_] (70) were investigated as potential catalysts for the catalytic reduction of N_2_O. Under 1 atm of N_2_O, 3.33 mol% of 62 in dimethylformamide led to >99% conversion of HBpin to O(Bpin)_2_ (Fig. 16a).^114^ The series of catalysts were then surveyed at 1 mol% loading and the arsenic clusters 65 and 70 were the only species to give >99% conversion under these conditions, suggesting that the identity of the [Pn_7_] core was the most significant aspect of the catalyst rather than the Group 13 moiety. Following this result, the K_3_Sb_7_ and K_5_Bi_4_ phases were investigated and afforded conversions of 38% and 45%, respectively. Interestingly, PPh_3_, AsPh_3_, KPPh_2_, and a mixture of B(C_6_F_5_)_3_ + P^*t*^Bu_3_, could not catalyse or very inefficiently catalysed the reduction of N_2_O, confirming that [Pn_7_] clusters can access catalytic cycles that classic FLPs and simple mono-pnictogen molecules cannot.

**Fig. 16:**
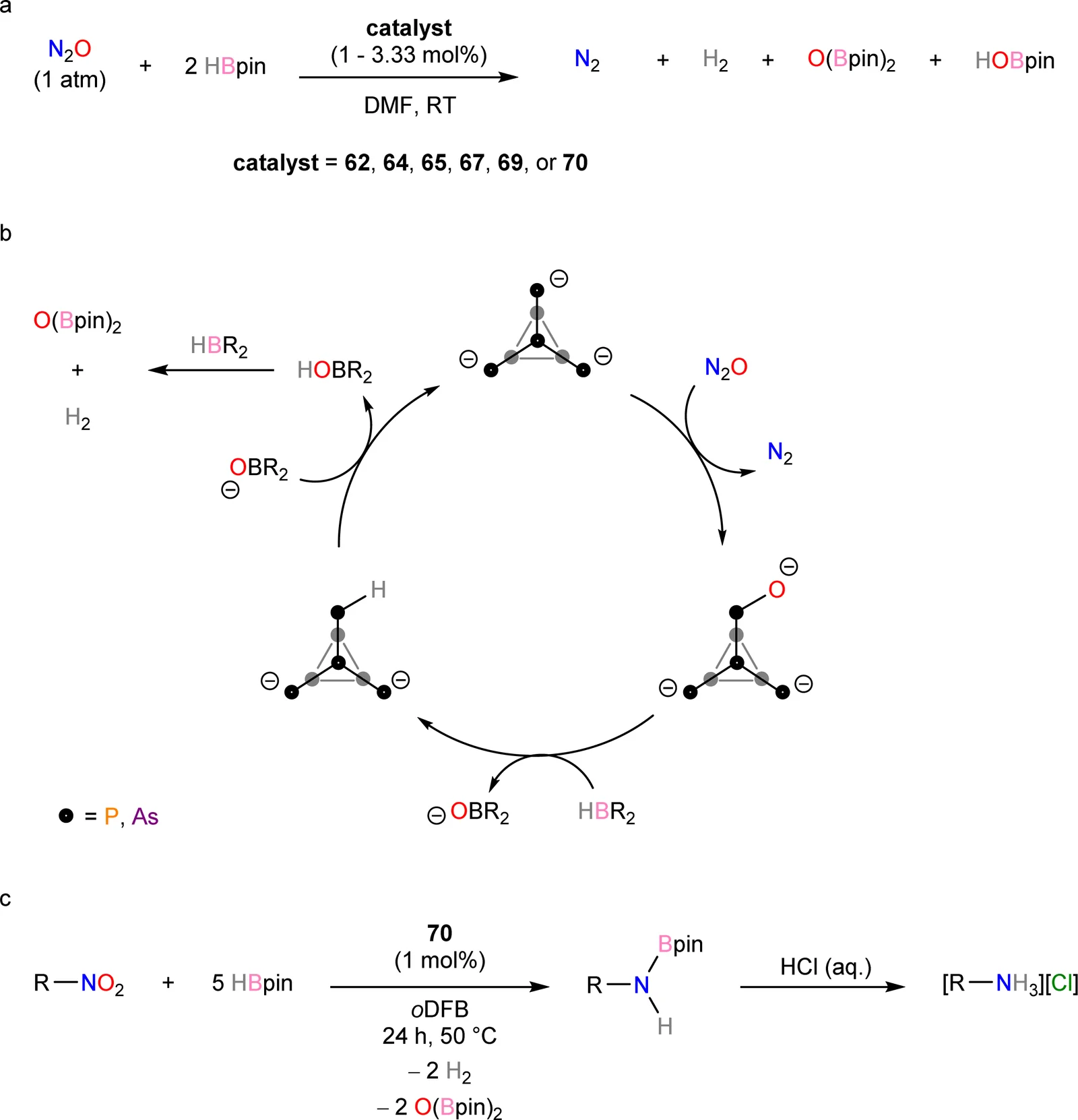
(a) Catalytic reduction of N_2_O by transition metal-free Zintl cluster catalysts. (b) Proposed mechanism for the reduction of N_2_O catalysed by [Pn_7_]^3−^ clusters. (c) Reduction of nitro compounds catalysed by 70.

Experimental and computational investigations were consistent with a proposed catalytic cycle involving initial O-atom transfer from N_2_O to the cluster, with the liberation of N_2_ (Fig. 16b). The oxidised cluster is then susceptible to reduction by the hydroborane to form [HPn_7_]^2−^, which is deprotonated by the generated oxyborane to regenerate the starting [Pn_7_]^3−^ cluster. The hydroxyborane then reacts with a second equivalent of hydroborane resulting in the elimination of H_2_ and the generation of the coupled R_2_BOBR_2_ species. This reactivity, therefore, represents a highly unusual case of Pn(–1/+1) redox cycling at the bridging pnictogen atoms at the [Pn_7_]^3−^ cluster. The more general utility of this reactivity was demonstrated in the stoichiometric oxidation of elemental sulfur, SO_2_, and aryldisulfide with [As_4_O_7_]^2−^, generated from the reaction of [As_7_]^3−^ with N_2_O. Furthermore, cluster 70 could also catalyse the hydroboration of nitro-functionalised compounds to afford amines with excellent efficiency (Fig. 16c).

## Outlook

Although catalytic applications of Zintl clusters have developed rapidly over the past decade, the field remains in its infancy compared with molecular main group and transition metal catalysis. Nevertheless, the examples highlighted throughout this Perspective demonstrate that Zintl clusters possess several characteristics that distinguish them from conventional catalyst platforms and suggest significant opportunities for future development.

1. Fluxional behaviour has traditionally been regarded as a complicating factor in the structural characterisation of Zintl clusters. However, recent catalytic studies increasingly suggest that dynamic rearrangements of the cluster framework can facilitate substrate binding, ligand exchange, and cooperative bond activation. Future catalyst design should seek to exploit, rather than suppress, controlled fluxionality as a catalytic design principle.

2. A second opportunity lies in the deliberate design of cooperative catalytic architectures. To date, Zintl clusters have successfully operated as transition metal supports, innocent platforms for appended catalytic functionalities, and non-innocent components that actively participate in substrate activation. However, the installed catalytic sites studied thus far operate independently even though these clusters often contain multiple binding sites that could be exploited to combine multiple different catalytic centres. Future developments in using these clusters as a platform for multimetallic and combined main group-transition metal co-operative catalysis are still needed.

3. While reversible cluster-centred redox processes underpin many aspects of Zintl chemistry, only very recently in the N_2_O work has a catalytic transformation involving a redox couple been reported. The reversible electron transfer chemistry of these clusters is yet to be fully harnessed, either by retaining the cluster framework between electron transfer processes or by moving between different cluster morphologies. It would be particularly exciting if multi-electron processes could be accessed here that go beyond two electrons.

4. Finally, the opportunity for Zintl clusters to act as a molecular bridge to heterogeneous catalysis must be realised. Their atomically precise structures permit mechanistic interrogation using techniques that are difficult to apply to conventional heterogeneous catalysts, while their structural relationship to extended solids provides valuable opportunities to translate molecular-level understanding into catalyst design. As synthetic methodologies continue to expand the accessible range of cluster compositions and functionalisation strategies, the transition from isolated homogeneous proof-of-concept studies towards predictive heterogeneous catalyst design is likely to define the next stage in the development of Zintl catalysis.

## Author contributions

B. v. I., J. S. W. and M. M. co-wrote the manuscript.

## Conflicts of interest

There are no conflicts to declare.

## Data Availability

There is no new data generated in this Perspective Article.
